# Determination and evaluation of the nonadditivity in wetting of molecularly heterogeneous surfaces

**DOI:** 10.1073/pnas.1916180116

**Published:** 2019-12-02

**Authors:** Zhi Luo, Anna Murello, David M. Wilkins, Filip Kovacik, Joachim Kohlbrecher, Aurel Radulescu, Halil I. Okur, Quy K. Ong, Sylvie Roke, Michele Ceriotti, Francesco Stellacci

**Affiliations:** ^a^Institute of Materials, École Polytechnique Fédérale de Lausanne, 1015 Lausanne, Switzerland;; ^b^Institute of Bioengineering, École Polytechnique Fédérale de Lausanne, 1015 Lausanne, Switzerland;; ^c^Laboratory for Neutron Scattering and Imaging, Paul Scherrer Institut, 5232 Villigen, Switzerland;; ^d^Forschungszentrum Jülich Gmbh, Jülich Center for Neutron Science at Heinz Maier-Leibnitz Zentrum, 85747 Garching, Germany

**Keywords:** hydration, hydrophobic, wetting, nanostructured

## Abstract

The interface between water and folded proteins is very complex. Proteins have “patchy” solvent-accessible areas composed of domains of varying hydrophobicity. The textbook understanding is that these domains contribute additively to interfacial properties (Cassie’s equation, CE). An ever-growing number of modeling papers question the validity of CE at molecular length scales, but there is no conclusive experiment to support this and no proposed new theoretical framework. Here, we study the wetting of model compounds with patchy surfaces differing solely in patchiness but not in composition. Were CE to be correct, these materials would have had the same solid–liquid work of adhesion (*W*_*SL*_) and time-averaged structure of interfacial water. We find considerable differences in *W*_*SL*_, and sum-frequency generation measurements of the interfacial water structure show distinctively different spectral features. Molecular-dynamics simulations of water on patchy surfaces capture the observed behaviors and point toward significant nonadditivity in water density and average orientation. They show that a description of the molecular arrangement on the surface is needed to predict its wetting properties. We propose a predictive model that considers, for every molecule, the contributions of its first-nearest neighbors as a descriptor to determine the wetting properties of the surface. The model is validated by measurements of *W*_*SL*_ in multiple solvents, where large differences are observed for solvents whose effective diameter is smaller than ∼6 Å. The experiments and theoretical model proposed here provide a starting point to develop a comprehensive understanding of complex biological interfaces as well as for the engineering of synthetic ones.

## Significance

Every folded protein presents an interface with water that is composed of domains of varying hydrophilicity/-phobicity. Many simulation studies have highlighted the nonadditivity in the wetting of such nanostructured surfaces in contrast with the accepted theoretical formula that is additive. We present here an experimental study on surfaces of identical composition but different organization of hydrophobic and hydrophilic domains. We prove that the interfacial energy of such surfaces differs by ∼20% and that a significant difference in the interfacial water H-bonding structure can be measured. As a result, in combination with molecular-dynamics simulations, we propose a model that captures the wetting of molecularly heterogeneous surfaces, showing the importance of local structure (first-nearest neighbors) in determining the wetting properties.

It is well-established that a large number of protein properties derive from their solvent-accessible surface (1). It is also known that such surfaces are highly structured with patches of varying composition and hydrophobicity. Such complexity affects a number of interfacial properties ranging from the work of adhesion [*W*_*SL*_, the main component in the interfacial energy (2)] to the very complex time-averaged structure of interfacial water (TASIW) (3). Whether these properties (as well as many others such as hydrophobic forces) are additive or not is a matter of intense debate (4–7). Classical thermodynamics for multicomponent interfaces treats *W*_*SL*_ additively. For example, one rigorous formulation for Cassie’s equation in the case of a perfectly flat surface with 2 components A and B is the following:

$$W_{SL}=f_{A}W_{A}+f_{B}W_{B}.$$ [1]

Several computational studies have appeared recently suggesting strong effects of patchiness on the density of interfacial water (typically used in simulation as a proxy for *W*_*SL*_) that cannot be explained in the framework of Eq. **1** (7–11). To the best of our knowledge, there is no direct experimental evidence supporting these claims. Some of us have used indirect observations (i.e., nonmonotonic dependence of *W*_*SL*_ on surface composition) to substantiate the existence of a geometrical effect in *W*_*SL*_ (12). Recently, experimental evidence of related physical phenomena has been published; Abbott and coworkers showed structural and chemical effects on hydrophobic forces (13), while Aida and coworkers showed local effects on ion pairing (14).

In the experimental cases discussed above, self-assembled monolayers (SAMs) composed of a mixture of hydrophobic and hydrophilic molecules were used as model surfaces. None of these studies could investigate one of the true key components of these interfaces, the TASIW. This is unfortunate, as it is the TASIW that is studied in all simulations. Unfortunately, probing the effect of patchiness on TASIW is a significant challenge when model systems (as well as proteins) differ both in composition and geometrical parameters, as it is impossible to decouple effects due to one or the other. Hence, there is no experimental evidence on the role of patchiness on TASIW. Here, to tackle this question, we have developed model compounds, i.e., gold nanoparticles coated with SAMs composed of a binary mixture of hydrophobic and hydrophilic ligands that separate into patches (15, 16). These model nanoparticles were produced in 2 forms differing solely in the geometrical parameters of the patches, but not in composition or any other structural parameters.

We used nanoparticles coated with a 1:1 binary mixture of perdeuterated 3-mercaptopropionic acid (dMPA) and 1-octanethiol (OT). These nanoparticles were chosen as they form patchy ligand shells, due to the fact that MPA is hydrophilic and OT hydrophobic, and that the longer OT gains conformational entropy at the edges of the patches. Also, they were expected to have nonadditive *W*_*SL*_ as they have been shown to have nonmonotonic solubility in various solvents (17). Similar nanoparticles have shown patches in their ligand shell that, at room temperature, did not change for days, but could be evolved into smaller patches by gentle heating (18). We started by exchanging, at room temperature, MPA ligands onto the ligand shell of OT nanoparticles. The exchange was tuned to lead to a 1:1 ligand shell composition (18). The ligand-shell composition was determined by NMR studies after etching the gold core. The resulting nanoparticles were then heated at 70 °C for 4 h, in order to bring the ligand-shell morphology to a different state. All of the detailed synthesis and characterization procedures are described in *SI Appendix*.

**Fig. 1.**
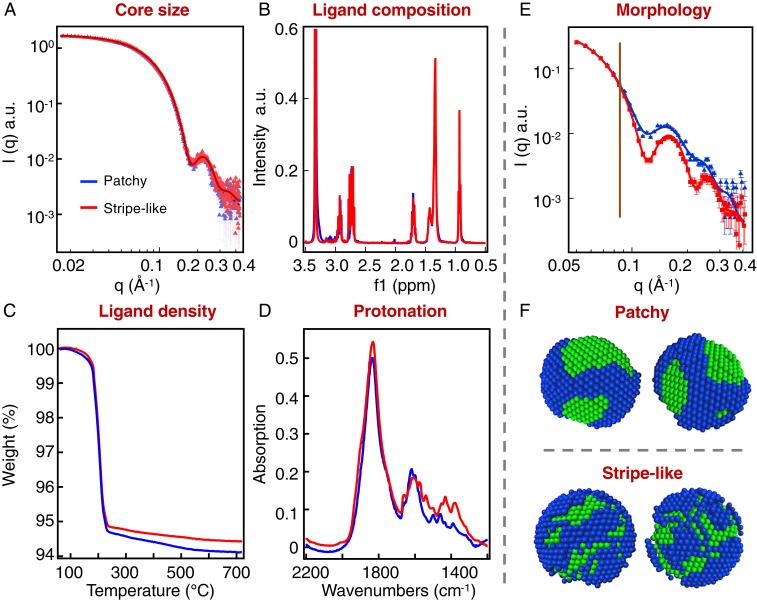
Characterization of the nanoparticles with OT and per-deuterated MPA used in this paper. In all plots the blue and red lines refer to nanoparticles before and after thermal treatment, respectively. Note for all measurements but SANS the curves practically overlap. (*A*) SAXS data on both nanoparticles were measured using ethanol as solvent. A fit with a Gaussian distribution gives a core size of 4.9 ± 0.6 nm, consistent with transmission electron microscopy (TEM) data shown in *SI Appendix*, Fig. S1. (*B*) NMR spectra of the ligands detached from their core via an iodine decomposition (22). The measured ligand ratio is approximately MPA: OT = 55%:45%. NMR spectra before decomposition are shown in *SI Appendix*, Fig. S2. (*C*) TGA plots for both particles showing that the ligand density is unaffected by the thermal treatment. Note that in TGA the experimental error is considered to be larger than 5%. (*D*) Fourier-transform infrared spectroscopy (FTIR) measurements taken from nanoparticle film in attenuated total reflection (ATR) mode. The spectra show that MPA ligands are mainly deprotonated on both nanoparticles. (*E*) SANS spectra of nanoparticles taken in tetrahydrofuran-d8. The overlap in the low-q region (below 0.083 Å^−1^ corresponding to the overall size of the NP in real space, as indicated by the vertical line) shows that the 2 nanoparticles have the same radius of gyration. The scattering curves at higher q range are the only characterization that shows a significant difference for the 2 nanoparticles, indicating a difference in the shape of nanoparticles (i.e., patchiness of the ligand shell). (*F*) Low-resolution models for the nanoparticles obtained by fitting the curves shown in *D* and in chloroform-d (dots are the experimental measures, and lines are the fits) using a method described recently (20). Detailed fitting procedures are described in *SI Appendix*. In the SANS model, the blue beads stand for OT ligands while the green ones are MPA ligands. We should point out that the nanoparticles characterized in *B* and *C* are nanoparticles coated with OT and MPA instead of dMPA. These nanoparticles are identical to the ones described in the other panels as discussed in *SI Appendix*.

After thermal treatment, care was taken to prove that no changes had occurred to the nanoparticles and that only the ligand-shell morphology had changed. Small-angle X-ray scattering (SAXS) was performed on the nanoparticles. As shown in Fig. 1*A*, the SAXS curves for the nanoparticles before and after thermal treatment overlap to an impressive degree. SAXS probes almost exclusively the nature (size and polydispersity) of the gold cores of the nanoparticles, as contrast in X-ray scales with the atomic number. The overlap between the 2 curves indicates that the gold cores were not affected by the thermal treatment either in size or in polydispersity. This is particularly important to guarantee that the curvature of the surface is the same for the 2 sets of nanoparticles. We then focused on characterizing the ligand shell before and after thermal treatment. Some of the characterizations were performed on nanoparticles coated with MPA and OT (as opposed to dMPA and OT). This choice was imposed by the technique used, in the case of ^1^H NMR because of its incompatibility with deuterated molecules, while in the case of thermogravimetric analysis (TGA) because of the large amount of sample required. We assume that these particles are identical to the deuterated ones. They were all obtained by place exchange reactions that started from the same OT nanoparticles and we have previously shown that deuteration of ligands does not change the properties of the nanoparticles (19, 20). A complete discussion on this topic can be found in *SI Appendix*. As is common in nanoparticle characterization (21), the ligand-shell composition was determined by etching the gold cores after having made sure that no free ligand was in solution (i.e., no sharp peak in the NMR of the whole nanoparticles, *SI Appendix*, Fig. S2). Fig. 1*B* compares the 2 NMR spectra achieved in this way. No measurable difference in the relative integration of the peaks was found, indicating no change in the ligand-shell composition (in both cases the ligand ratio was found to be approximately MPA: OT = 55%: 45%). We performed TGA to determine ligand density on the nanoparticles. Fig. 1*C* shows that the 2 TGA curves are well within instrumental error of each other (the difference in the plateau is <2% in mass and the accepted instrumental error is >5%). This indicates that the ligand-shell density did not change. Given that the relative ratio of the ligands and the total density did not change, we can conclude that the average number of OT and of MPA molecules on the nanoparticles before and after thermal treatment are the same. Finally, infrared spectroscopy (Fig. 1*D*) measurements show that the protonation state of the carboxylic acid had not changed, as both spectra show only a peak at ∼1,600 cm^−1^ corresponding to the asymmetric stretching of deprotonated carboxylic group. Hence, we can conclude that the chemical composition of the ligand shell did not change upon thermal treatment. In *SI Appendix*, Table S1, we report a summary of all of the results of the characterization performed on these particles.

In striking contrast to what is described above, small-angle neutron scattering (SANS) led to significantly different scattering curves for the 2 nanoparticles (Fig. 1*E*); i.e., there are substantial differences in the scattering form factors of the nanoparticles. Indeed, the SANS of the thermally treated nanoparticles shows more pronounced oscillations, indicating a shape that is more centrosymmetric when compared to the untreated nanoparticles. Such changes in form factors, given all of the other measurements described above, can be assigned solely to differences in the overall shape of the nanoparticles. Given that the gold core had no change in form factor (as derived from SAXS), this means that the form factor change in SANS has to be attributed to a change in the shape of the ligand shell. The latter was proven not to change in chemical nature, so that the only remaining possibility is a change in the patchiness. Such effects of thermal treatment on the morphology of nanoparticles are reproduced at 2 different neutron scattering facilities, as shown in *SI Appendix*, Fig. S3, which confirms that the differences in the scattering profile are not due to instrumentation. Using a method recently reported (20), we were able to determine the morphology of the patches using SANS curves recorded with different solvent contrast and ab initio calculations. A detailed description can be found in *SI Appendix* and all of the SANS spectra utilized with their fitting are shown in *SI Appendix*, Fig. S4. The resulting models for the ligand shells show a patchy-type morphology for nanoparticles before thermal treatment and stripelike structure after thermal treatment, as presented in Fig. 1*F*. By quantifying the characteristic length scales of the patches from the SANS modeling (20), we find that the average MPA domain thickness is 2.1 ± 0.6 nm and 1.3 ± 0.3 nm for the patchy (untreated) and stripelike (thermally treated) nanoparticles respectively.

**Fig. 2.**
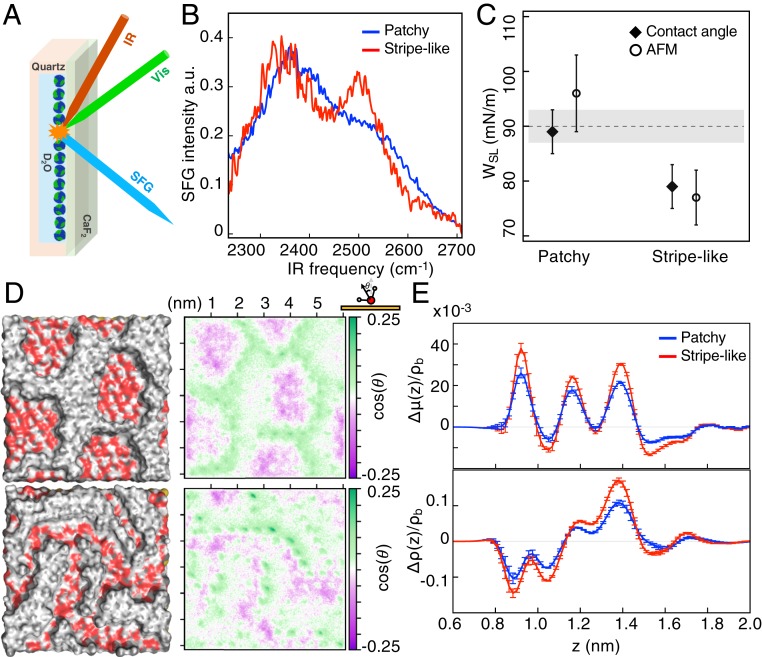
Experimental characterization and MD simulations of the water interfaces around the nanoparticles. (*A*) Schematic of the SFG setup. The SSP polarization is used to measure the interfacial hydrogen bond strength. (*B*) SFG spectra collected at the nanoparticle–water interface. The D-bond network has significantly higher fraction of weak bonds for stripelike nanoparticles. (*C*) Measurements of the *W*_*SL*_ for films of nanoparticles, obtained independently using 2 techniques, contact angle (black diamonds) and AFM (white circles). The measurements show large differences. The dotted line represents the additive averages for the *W*_*SL*_ of the 2 homoligand nanoparticles measured using contact angle. The gray band is the error in the measurements. (*D*, *Left*) Morphology of the surfaces in the simulation, after equilibration. Water molecules have been removed to show the geometry of the interface. Carbon, oxygen, and hydrogen atoms are represented with gray, red, and white colors respectively. (*D*, *Right*) Corresponding 2D histogram of the average dipole orientation density, μ, above the surfaces (averaged over 4 different independent simulations). Areas on top of the MPA patches look mostly purple (dipole pointing toward the surface) and areas on top of the OT patches look mostly white (dipole parallel to the surface). Green regions (dipole pointing out from the surface) are visible on top of the OT-MPA interfaces, with darker green visible on the stripelike morphology. (*E*) Deviation from the additive assumption of the average dipole orientation density, Δμ, (*Top*) and density profile of water, Δρ (*Bottom*), as a function of the distance from the gold surface (z).

Given that the shape of the nanoparticles and the chemical nature and composition of their ligand shell were the same, we were able to characterize the TASIW for both particles, as any difference found would be due solely to the different geometrical structure of the ligand shell. We used vibrational sum-frequency generation (SFG) to characterize the interfacial water structure as it is a surface-sensitive technique that has been proven to effectively characterize the vibrational spectra of interfacial water (23). Fig. 2*A* presents an illustration of an SFG experiment. Films of nanoparticles were produced on CaF_2_ surfaces by drop-casting ethanol nanoparticles solutions. Detailed procedures and parameters are described in *SI Appendix*. We first used SFG to characterize the C-H stretching peaks of the OT ligands in the 2 nanoparticles. As described in *SI Appendix*, Text and Fig. S5, the SFG spectra for these stretching modes for the 2 nanoparticles agree with the SANS models. Fig. 2*B* shows the interfacial water structure as measured in the O-D stretch region of the SFG spectrum; the spectra were found to differ considerably. The spectra have 2 main features, i.e., a peak at ∼2,375 cm^−1^ and a peak at ∼2,530 cm^−1^, corresponding to interfacial water that have stronger (2,375 cm^−1^) and weaker hydrogen bonds. It can be seen from Fig. 2*B* that the TASIW is different for the 2 nanoparticles. Water molecules at the interfaces with the 2 nanoparticles experience different intermolecular H-bond environments. In the case of water at the interface with the patchy nanoparticle, the 2,530-cm^−1^ band is mostly a shoulder of the 2,375-cm^−1^ band. For the case of the stripelike nanoparticle, the 2,530-cm^−1^ band has almost the same intensity as the 2,375-cm^−1^ band. To make sure that these effects were not due to film morphology but due to wetting at the single-nanoparticle level, multiple spectra were recorded on different films; the spectra are shown in *SI Appendix*, Fig. S6. The general conclusion is that in the case of stripelike structure the ratio of the 2,530-cm^−1^ band to the 2,375-cm^−1^ band is larger than that for the patchy nanoparticle. An oversimplified interpretation of these data is that water molecules are less structured near the stripelike nanoparticles than near the patchy nanoparticles.

We then went on to establish whether such differences would lead to different *W*_*SL*_. We first measured contact angles for films of the 2 nanoparticles and derived the *W*_*SL*_, as listed in *SI Appendix*, Table S2. In Fig. 2*C* we show that the *W*_*SL*_ for the patchy nanoparticles is close to the average of the *W*_*SL*_ of the 2 homoligand nanoparticles, while a deviation is found for the nanoparticles with stripelike morphology. To further confirm these data, we employed a recently developed technique (24) based on small-amplitude modulation atomic force microscopy (AFM) to measure the *W*_*SL*_ (additional information can be found in *SI Appendix*, Text and Fig. S7). Also, in this case we found a difference between the 2 samples (Fig. 2*C*). It should be stressed that this latter technique is based on different working principles than contact-angle measurements, and measures *W*_*SL*_ at the single-nanoparticle level, removing the effects of roughness on the measurement. The agreement between the 2 measurements strongly validates the conclusions. Averaging the results from the 2 independent techniques we can conclude that the difference between the *W*_*SL*_ on the 2 nanoparticles is 15 ± 7 mN/m (∼17% of the expected additive value), the stripelike nanoparticles being more hydrophobic. Additional results on a different set of nanoparticles are reported in *SI Appendix*, Table S3.

The experimental evidence we have provided thus far shows that the hydrophobicity of the nanoparticles depends on the morphology of the SAM in a nonadditive way, and that the effect is connected to the TASIW. In order to capture the relationship between surface morphology and TASIW structure, we performed large-scale molecular-dynamics (MD) simulations of a force-field model of SAMs composed of mixtures of OT and MPA (in its protonated form) together with water. We generated 2 configurations (Fig. 2*D*) that are consistent with the ligand distributions seen on the patchy and stripelike surfaces. It is found that water on top of the patchy and stripelike configurations exhibits a complex behavior, i.e., realistic arrangements of ligands do not lend themselves to a simple interpretation in terms of clear-cut molecular motifs. In Fig. 2*D* we show the time-averaged dipole orientation density of the water molecules at the interfaces. It is obvious that the 2 surfaces lead to different angular distributions. We notice that the water dipoles point toward the surface on top of the MPA patches and lie parallel to the surface on top of the OT patches. The dipoles point out from the surface at the interface between OT and MPA. We find a larger number of dipoles pointing out from the surface on the stripelike morphology because the MPA–OT interfaces are closer to each other. The integrated dipole orientation density μ together with the water density profile ρ for the 2 SAM geometries are then calculated to illustrate the SAM-induced water ordering. The theoretical μ and ρ profiles based on an additive assumption are also calculated by simulating pure OT and MPA SAM surfaces. The deviations of μ and ρ from the additive assumption are then plotted as a function of the position on the surface (Fig. 2*E*). One can see that the dipole and density profiles above both SAMs do not follow a purely additive behavior and that there are significant differences between the 2 morphologies. Data resulting from the simulation of 2 additional surfaces are reported in *SI Appendix*, Fig. S8 and show the high reproducibility of the obtained results.

**Fig. 3.**
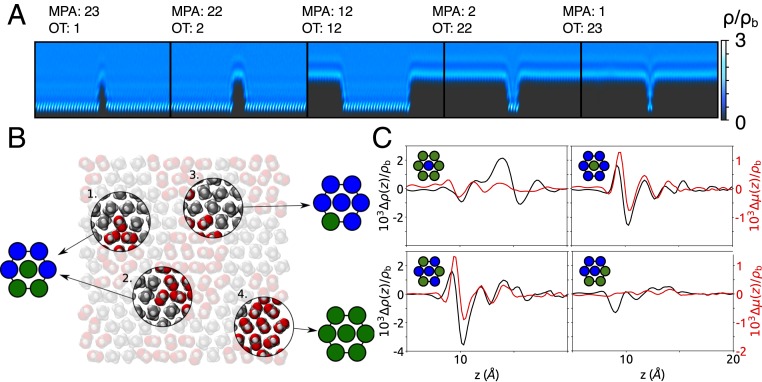
Water density on heterogeneous surfaces. (*A*) Water density in a cut across trenches of varying size. Ligands occupy the black area of the pictures (shorter ligands are MPA, longer ligands are OT). (*B*) Schematics of the nearest-neighbor classification scheme showing 4 ligands with their surrounding environments: patterns 1 and 2 are equivalent by rotational symmetry, with an MPA ligand at the center, surrounded by 2 other MPA ligands in an “*ortho*” configuration; pattern 3 has an OT ligand at the center with 5 other OT ligands surrounding it; pattern 4 has an MPA ligand surrounded by 5 other MPA ligands, and does not contribute to the excess term. (*C*) Example of surface excesses associated with some of the possible nearest-neighbor configurations.

In order to propose a quantitative model for these nonadditive effects, we began by performing MD simulations of a series of “trench” configurations corresponding to rectangular OT and MPA regions of varying width (*SI Appendix*, Fig. S9), where we expect that the boundary between the 2 types of ligand is responsible for nonadditive behavior. Fig. 3*A* shows the structuring of water in a cut across trenches of varying sizes, where it can clearly be seen that the pattern of the water density at the boundaries of the trenches is very similar for all trenches that are more than 1 molecule thick; for these narrower trenches clear differences are observed (a detailed description of the results can be found in *SI Appendix*, Text and Figs. S10 and S11). Specifically, the water density on a single MPA molecule, surrounded by OT, is smaller than the one on thicker MPA trenches. On the contrary, the water density on a single OT molecule, surrounded by MPA, is higher than the one on thicker OT trenches. Overall these observations suggest that the water density on a ligand is affected primarily by its first-nearest neighbors.

These simulations demonstrate that the nonadditivity in the systems containing trenches is due to the water structure at the boundary between 2 types of ligand. However, these systems contain only straight boundaries between ligands of different types, and do not provide an exhaustive list of possible nearest-neighbor configurations, limiting the power of a model based on trench systems to predict the results of a general surface. For this reason, we then generated a set of simulation boxes in which the ligands in each site on both the top and the bottom surfaces were chosen randomly. Assuming that the contribution of a particular ligand to nonadditivity depends only on the identities of its first-nearest neighbors, and treating the long-range structure as a mean field, each ligand on a surface was classified according to the identities and dispositions of its first-nearest neighbors. Fig. 3*B* illustrates the definition of a particular ligand’s environment, and *SI Appendix*, Text and Fig. S12 describe the classification of these ligands. Using the excess terms Δμ and Δρ for these randomly generated surfaces, we built a linear regression model, in which an excess quantity for an arbitrary surface is predicted as

$$\Delta f\left(z\right)=\sum n_{i}f_{i}\left(z\right),$$ [2]

where *f* = μ or ρ, *n*_*i*_ is the number of ligands of type *i*, and *f*_*i*_(*z*) is a function to be fitted. This is analogous to the cluster expansion models that are used, e.g., to describe the thermodynamic properties of multicomponent alloys (25, 26). Here, we use this approximation to model excess terms associated with a particular distribution of ligands. Fig. 3*C* shows the excess terms associated with some of the possible nearest-neighbor arrangements (the full set of possibilities is shown in *SI Appendix*, Fig. S12). These examples have been chosen to show 2 effects. The first of these is asymmetry between the 2 ligand types: the excess term associated with a single MPA molecule surrounded by 6 OT molecules is significantly different from that associated with 1 OT molecule surrounded by 6 MPA molecules. The second is that the excess terms are not determined only by the number of nearest neighbors of a certain kind but also on their arrangement (with “patchy” patterns usually being associated with smaller excesses).

**Fig. 4.**
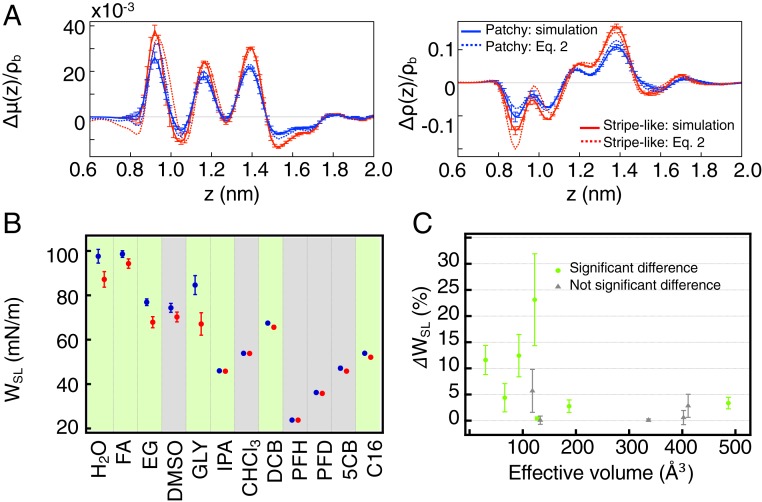
Model capturing the nonadditivity of interface-dependent properties. (*A*) Surface excess Δρ and Δμ of the patchy and stripelike surfaces as calculated from MD simulations and calculated by the linear regression model. (*B*) *W*_*SL*_ values of the patchy (blue) and stripelike (red) nanoparticles in various solvents. The green shadings indicate solvents in which the *W*_*SL*_ shows significant differences (Student’s *t* test assuming equal variances, *P* = 0.05) for the 2 nanoparticles. The gray shadings indicate that the null hypothesis (“the 2 nanoparticles have the same work of adhesion”) cannot be rejected. [The solvents used are the following: water (H_2_O), formamide (FA), ethylene glycol (EG), dimethyl sulfoxide (DMSO), glycerol (GLY), isopropanol (IPA), chloroform (CHCl_3_), dichlorobenzene (DCB), perfluorohexane (PFH), perfluorodecalin (PFD), 4-Cyano-4′-n-pentylbiphenyl (5CB), and hexadecane (C16).] (*C*) Dependence of the differences of *W*_*SL*_ $\left(\Delta W_{SL}\right)$ of the 2 films on the effective volume of the solvent molecules. The color of the points is the same as the one of the shadings in ref. 3 and relates to the significance of the differences in *W*_*SL*_.

Fig. 4*A* shows the predictions of this model for the surface excesses of the patchy and striped systems. The excellent match between the excess values resulting from the simulations (continuous lines) with the ones resulting from the predictions derived from Eq. **2** (dotted lines) indicates that the first-nearest neighbors are sufficient to determine the excess term associated with a single molecule in the SAM. Therefore, we expect the work of adhesion between the SAM and a solvent not to be affected by the surface heterogeneity when the solvent molecules are larger than the intermolecular distance in the SAM (because a single solvent molecule will interact with more than 1 molecule on the surface at any time). To test this prediction, we measured *W*_*SL*_ in films of the nanoparticles described above for a series of solvents. Every time a film was produced, its contact angle with water was measured, to make sure the roughness was homogeneous. All of the contact angle data as well as the solvent properties are listed in *SI Appendix*, Table S4. As shown in Fig. 4*B*, some solvents have the same *W*_*SL*_ for the patchy and stripelike nanoparticles, while others behave like water, featuring different *W*_*SL*_. To rationalize these results along the lines of Eq. **2**, we calculated the effective molecular volume (V_eff_) of all of the solvents by dividing their molecular weight by the product of the solvent density and the Avogadro number. We plotted the differences in *W*_*SL*_ $\left(\Delta W_{SL}\right)$ against V_eff_ and the results were striking (Fig. 4*C*). Solvents whose V_eff_ is above ∼125 Å^3^ (corresponding to an effective diameter of ∼6 Å, comparable with the intermolecular distance on the SAM: ∼5 Å) show a $\Delta W_{SL}$ that is almost independent of V_eff_ and smaller than 5%. Solvents with V_eff_ below ∼125 Å^3^ have larger $\Delta W_{SL}$ with large variations among them. These results confirm the predictive power of our model. We have tried to correlate the large variations at low V_eff_ to the properties of the solvents we used, to no avail. The most likely explanation is that the work of adhesion is affected by many properties of the solvent (such as its polarity, polarizability, etc.) but thus far we could not find a single predictive property for its magnitude. To further confirm our hypothesis on the size of the molecules of the solvents, we have reviewed previously published data (17), where the saturation concentration of nanoparticles similar to the ones studied here was evaluated in a large number of solvents. It was reported that a nonmonotonic dependence of the saturation concentration on the ligand-shell composition was observed only for a subset of solvents while another subset provided solubility data that could be simply explained in terms of average hydrophobicity of the ligand shell of the nanoparticles. The paper (17) provided no explanation on whether a solvent was part of one or the other subset. Reevaluating those data, we find that the subset of solvent that showed a nonmonotonic dependence on ligand-shell composition is composed of solvents whose V_eff_ is smaller than ∼130 Å^3^, in agreement with our hypothesis (*SI Appendix*, Fig. S13).

The theoretical construction, supported by the experiments and simulations that we have reported here, represents an important step forward toward a molecular understanding of wetting. It must be seen as an attempt to challenge the currently accepted paradigm that considers concepts like hydrophobicity as absolute. Extending these findings to more complex scenarios, such as biological systems and proteins, will require significant extensions to the experimental and theoretical approach, e.g., to account for the dynamical nature of peptides. The concepts and techniques we introduced here, however, lay the foundations for these studies, and for a deeper understanding of the interplay between the chemical nature and the geometric arrangement of molecular units at interfaces.

## Materials and Methods

### Chemicals.

Chloro(triphenylphosphine)gold(I) (≥99.9% trace metals basis, Aldrich), 1-Octanethiol (≥98.5%, Aldrich), 3-Mercaptopropionic acid (HPLC, ≥99.0%, Aldrich), 3-Mercaptopropionic-2,2,3,3-d4 Acid (98 atom % D, C/D/N Isotopes Inc.), borane t-butylamine complex (97%, Aldrich), Acetone(HPLC, ≥99.8%, Aldrich), Methanol (HPLC, ≥99.9%, Aldrich), Ethanol (HPLC, ≥99.8%, Aldrich), Chloroform (HPLC, ≥99.9%, Aldrich), Toluene (HPLC, 99.8%, Aldrich), Sulfuric acid (98.0%, Aldrich), (3-Mercaptopropyl)trimethoxysilane (95%, Aldrich), Hexane (99%, Aldrich), Iodine (≥99.99% trace metals basis, Aldrich), Tetrahydrofuran-d8 (≥99.5 atom % D, Aldrich), Chloroform-d (99.8 atom % D, Aldrich), Methanol-d4 (99.96 atom % D, Aldrich). All chemicals were used as received.

### Synthesis of OT-Protected Gold Nanoparticles.

The OT homoligand-protected nanoparticles were synthesized following a modified protocol of Stucky and coworkers. Chloro(triphenylphosphine)gold(I) (123 mg) was dissolved in 40 mL 1:1 mixture of chloroform and toluene. OT ligand (0.25 mmol) was added to the solution. The mixture was heated to 70 °C for 15 min. Then, 217 mg borane t-butylamine complex was added quickly into the solution under stirring. The color of the solution changed from brown to dark red. After 1 h of reaction, 40 mL methanol was added to quench the reaction and the solution was cooled down to room temperature. Nanoparticles were precipitated as black powder and purified by repeated centrifugation using acetone and methanol. The final black precipitates (∼40 mg) were dried under vacuum overnight.

### Synthesis of MPA-OT Mixed-Ligand Gold Nanoparticles by Ligand Exchange Reaction.

OT homo-ligand protected nanoparticles (40 mg) were dissolved in 20 mL chloroform. MPA (2.3 μL) was added to the solution under vigorous stirring. After 6 h of reaction at room temperature, toluene (20 mL) was added to precipitate the nanoparticles. The precipitate was washed multiple times using chloroform and toluene, and then dried under vacuum overnight.

### Thermotreatment of MPA-OT Mixed-Ligand Nanoparticles.

MPA-OT (20 mg) nanoparticles were dissolved in 5 mL ethanol (containing 0.01% vol/vol sulfuric acid). The solution was heated at 70 °C under stirring for 4 h before being cooled down to room temperature. Then the nanoparticles were precipitated by adding 30 mL hexane and washed using chloroform before being dried under vacuum overnight.

### SANS.

SANS measurements were conducted on the SANS-I instrument at Paul Scherrer Institute and further confirmed on the KWS-2 instrument at Jülich Center for Neutron Science. For both measurements, a nanoparticle solution at a concentration of 10 mg/mL was used corresponding to a volume fraction of less than 0.1%. Measurements at SANS-1 were performed at 20 °C, using 1.5-m sample-to-detector distance, at 5-Å wavelength with a collimation setup of 6 m covering a q range from 0.03 to 0.5 Å^−1^. Similarly, measurements on KWS-2 were performed at 20 °C, using 1.7-m sample-to-detector distance, at 4.7-Å wavelength with a collimation setup of 8 m and a q range from 0.03 to 0.5 Å^−1^. The data acquisition time was 2 h for each sample. The 2D scattering data were processed and reduced using BerSANS or QtiKWS software for the radial averaging, background subtraction, transmission correction, and normalization to the absolute scale.

### SAXS.

SAXS measurements were performed using Rigaku BioSAXS 2000. Both nanoparticles were dissolved in ethanol (containing 0.1% H_2_SO_4_ to protonate the MPA ligands in order to gain better colloidal stability) with a concentration of ∼0.1 mg/mL. Soda lime glass capillaries with inner diameter 1.0 mm and wall thickness of 0.01 mm were used for the measurements. The data acquisition time is 2 h for each sample. The SAXS data were fitted using SasView software by a core-shell sphere model assuming a Gaussian distribution.

### TEM.

TEM images were taken using an FEI Tecnai Osiris instrument at an accelerating voltage of 120 kV. The nanoparticle samples were prepared by drop-casting 2 μL of the above-mentioned ethanol solution (0.1 mg/mL) onto the carbon-coated-copper 400 mesh grid following by drying under ambient condition.

### NMR.

NMR spectra were recorded using a Bruker 400MHz spectrometer. In order to determine the ligand ratio on nanoparticle surfaces, 5 mg nanoparticles were first dissolved in 0.5 mL deuterated methanol solvent and ^1^H NMR spectra were then taken to make sure that no sharp peaks corresponding to unbounded small organic molecules exist. Then 0.1 mL deuterated methanol solution of iodine with a concentration of 50 mg/mL was added into the nanoparticle solution to etch the core of nanoparticles. After the etching of the gold core, the solution is filtered and ^1^H NMR spectra were taken again to determine the ligand ratio on nanoparticle surfaces.

### TGA.

TGA measurements were done using a TGA 4000 instrument from Perkin-Elmer. Around 15 mg of nanoparticle samples were used. The glow of nitrogen gas is at 20.0 mL/min. The samples were heated from 50 to 750 °C, at 5.0 C/min.

### FTIR.

The 6700 Nicolet instrument from Thermo Fischer Scientific was used for measuring the infrared (IR) spectra. Nanoparticle ethanol solution of 10 mg/mL was drop-casted on the ATR crystal and dried completely. A drop of water was added on top of the crystal. 512 spectra were taken at 2-cm^−1^ resolution and averaged for each sample. Baseline correction was performed using the OMNIC software.

### Contact Angle.

Glass slides were first repeatedly cleaned using ethanol and acetone to remove surface contaminants. Then they were immersed in freshly prepared base solution, sonicated for 3 min, and rinsed with deionized water and ethanol before being dried under vacuum. The film of nanoparticles (formed at the water–air interface) was deposited on the glass slide. The procedure was repeated 3 times, letting the film dry in between consecutive depositions. The slides were then completely dried under vacuum before the contact angle measurements. Contact angles were measured using DataPhysics OCA 35 Instruments. More than 10 drops of 5 μL were deposited on different sites of the substrates.

For contact angle measurements of different organic solvents, the water contact angle of the nanoparticle films was first measured to make sure that the same results were reproduced, i.e., the films have the same roughness. For each solvent, at least 3 drops of 2 μL were deposited on different sites of the substrates. All of the measured contact angle data as well as the solvent properties are listed in *SI Appendix*, Table S4.

### SFG.

The sample preparation was performed as described below: the CaF_2_ window was cleaned with ethanol and ultrapure water. The nanoparticle ethanol solution (prepared as described above, 0.1 mg/mL) was drop-casted on top of the CaF_2_ window. After solvent evaporation the window was finally flushed with argon, just before the measurement. 25 µl of D_2_O were added on top of the nanoparticle film in a sample cuvette with a path length of 0.2 mm.

Vibrational SFS spectra were recorded using the setup for SFG experiments described in ref. 23. An 800-nm regeneratively amplified Ti:sapphire system (Spitfire Pro, Spectra Physics) seeded with an 80-MHz, 800-nm oscillator (Integral 50, Femtolasers) was operated at a 1-kHz repetition rate to pump a commercial OPG/OPA/DFG (optical parametric generation/optical parametric amplifier/difference frequency generation) system (HE-TOPAS-C, Light Conversion), which was used to generate IR pulses. The visible beam was split off directly from the amplifier, and spectrally shaped with a home-built pulse shaper. The angle between the 10-mJ visible (VIS) beam [800 nm, full width at half maximum (FWHM) 15 cm] and the 6-mJ IR beam (3–4.5 mm, FWHM 160 cm) was 20 (as measured in air). The focused laser beams were overlapped in the sample cuvette. The reflected SF light was collimated using a planoconvex lens (f.15 mm, Thorlabs LA1540-B) and passed through 2 short-wave pass filters (Third Millennium, 3RD770SP). The SF light was spectrally dispersed with a monochromator (Acton, SpectraPro 2300i) and detected with an intensified CCD camera (Princeton Instruments, PI-Max3) using a gate width of 10 ns. The acquisition time for a single spectrum was 150 s. A Glan–Taylor prism (Thorlabs, GT15-B), a half-wave plate (EKSMA, 460–4215), a polarizing beam-splitter cube (CVI, PBS-800-050), and 2 BaF2 wire grid polarizers (Thorlabs, WP25H-B) were used to control the polarization of the SFG, VIS, and IR beams, respectively.

All measurements were performed in the SSP (s-polarized sum frequency, s-polarized visible and p-polarized infrared beams) polarization combinations. The optical path was closed in a box filled with nitrogen, in order to reduce absorption from CO_2_. The collection of the full spectra was achieved by combining multiple spectra collected shifting the frequency of the IR beam by 200 cm^−1^ every time.

### AFM.

The sample preparation for AFM is similar to the one for contact angle described above except for the fact that the glass slides were functionalized before the deposition of the nanoparticles: 50 mM (3-mercaptopropyl)trimethoxysilane acetone solution was used to immerse the slides for 4 h in order to functionalize the surface. The glass surfaces were then rinsed multiple times with ethanol and deionized water and the nanoparticle film was deposited as described above. The samples were then completely dried under vacuum. Multiple glass slides were attached to the same metal disk (TED PELLA) using the wax Apiezon 100. In this way, it was possible to move from one sample to the other during the experiment without unmounting the sample cell in the AFM.

AFM images were collected in amplitude modulation mode on a commercial Cypher ES system (Asylum Research/Oxford Instruments). The temperature was kept constant at 25 °C during all of the measurements. The sensitivity of the cantilevers (RC800 PSA, lever number 1, Olympus) was evaluated from force curves and the spring constant was measured from their thermal spectra. The cantilevers were driven acoustically. Typical scan rates were in the range 5–13 Hz.

The values of the free amplitude and free phase of oscillation were extracted from dynamic force curves collected immediately after high-resolution imaging was achieved.

### Simulation Details.

All simulations were carried out using the GROMACS/2018.4 software package (27), with the OPLS/AA force field (28) used to describe interligand interactions, the SPC/E model (29) for water and the GolP model for the gold surface (30). In all simulations, a gold slab with height 5 nm is placed at the center of the simulation box, ligands attached to the surface (with the positions of the sulfur atom of each ligand fixed throughout each simulation, as were all gold atoms other than the image-charge sites) (30) and 5-nm slabs of water placed above and below the gold surface and allowed to condense onto the ligands. Further details of the setup and running of the simulations are given in *SI Appendix*.

The structure of the solvent around the nanoparticle surface was quantified using 2 properties: the density ρ, obtained by taking a histogram of coordinates of the oxygen atoms in water molecules, and the dipole orientation density μ = <cosθ>ρ, where <cosθ> is the average orientation of the water dipole moment with the surface normal pointing away from the gold surface.

## Supplementary Material

Supplementary File
