# Supplementary material for: Determination and evaluation of the nonadditivity in wetting of molecularly heterogeneous surfaces
Source: Proc Natl Acad Sci U S A. 2019 Dec 2;116(51):25516–23. doi: 10.1073/pnas.1916180116 (PMC6926055; doi:10.1073/pnas.1916180116)
Supplement: Supplementary File [file pnas.1916180116.sapp.pdf]

PNAS
www.pnas.org

Supplementary Information for

Determination and Evaluation of the Non-Additivity in Wetting of Molecularly Heterogeneous Surfaces

Zhi Luo,[1]† Anna Murello,[1]† David M. Wilkins,[1]† Filip Kovacik,[2] Joachim Kohlbrecher,[3] Aurel Radulescu,[4] Halil I. Okur,[2] Quy K. Ong,[1] Sylvie Roke,[1,2] Michele Ceriotti,[1] Francesco Stellacci[1,2]*

[1]Institute of Materials, École Polytechnique Fédérale de Lausanne, 1015 Lausanne, Switzerland.

[2]Institute of Bioengineering, École Polytechnique Fédérale de Lausanne, 1015 Lausanne, Switzerland.

[3]Laboratory for Neutron Scattering and Imaging, Paul-Scherrer Institut, 5232 Villigen, Switzerland.

[4]Forschungszentrum Jülich Gmbh, Jülich Center for Neutron Science, JCNS at Heinz Maier-Leibnitz Zentrum, Lichtenbergstraße 1, 85747 Garching, Germany.

†These authors contributed equally to this work.

*Correspondence to: francesco.stellacci@epfl.ch

This PDF file includes:

Supplementary text
Figures S1 to S13
Tables S1 to S4
SI References

www.pnas.org/cgi/doi/10.1073/pnas.1916180116

Supplementary Text

Effect of deuteration on the ligand shell nanoparticles

In the current work, most of the data i.e. SANS, SAXS, FTIR, TEM, SFG, AFM, and Contact angle, were measured on the same batches of nanoparticles, i.e. NPs coated with dMPA and OT. For the measurements of NMR and TGA, we used NPs coated with MPA and OT due to the requirements of the techniques. All the nanoparticles were synthesized using a ligand exchange reaction from the same batch of OT homoligand NPs. Therefore, they should have the same core size and size distribution. For the ligand shell composition, we have previously shown that the deuteration does not affect the ligand shell composition for the same(1) and similar(2) type of ligand coated NPs. Furthermore, while for the SANS modelling, only 50% penalty was imposed on the volume fraction of the ligands, i.e. the ligand shell ratio was not fixed. The volume ratio of the outcome model is MPA: OT = 1: 2.12. While the ligand ratio from NMR measurements is MPA: OT= 55%: 45%, which corresponds to approximately MPA: OT= 1: 2.18, agreeing well with the resulted 3D model. The good quality of the fits also confirms that the ligand ratios are consistent with NMR data.

Ab initio model fitting of SANS data

As described in a recent report, the construction of 3D models from SANS data was performed using MONSA software(3). Briefly, a spherical search volume with a diameter of 7.2 nm was generated according to the pair distribution function calculated using GNOM(4) program. The search volume composed of closely packed beads with a radius of 2 Å. Each bead could be assigned either to the gold, MPA, OT or solvent phase. To facilitate the calculation, the beads within 2 nm radius are fixed to be gold, while beads between 2.7 nm 3.6 nm radius could be assigned to either the two types of ligands or solvent. The ratio of volume fraction of the two types of ligands, calculated from the NMR and TGA value (OT: MPA= 2.0: 1), was used as a fitting penalty during the simulated annealing process. Starting from random configurations, simulated annealing was conducted in order to minimize the discrepancy between the experimental data and the form factor of the bead model. The discrepancy is defined as:

$$\chi^2 = \sum_k \frac{1}{N_k - 1} \sum_{j=1}^{N_k} \left[\frac{I_{\text{exp}}^{(k)}(q_j) - c_k I_{\text{calc}}^{(k)}(q_j)}{\sigma^{(k)}(q_j)} \right]^2$$

Where, the k is the index of scattering curves; N_k is the number of points in the experimental scattering curves; $I_{exp}(q)$ and $I_{calc}(q)$ are the intensities from the experiments and the calculation of the bead model; c_k is the scale factor and $\sigma(q)$ is the experimental errors at each q.

The model fitting process was repeated multiple times always starting from random configurations and the resulting models all present similar ligand shell organization features.

Interpretation of the C-H region of SFG spectra

Since the morphological evolution during the annealing is driven by the gain in conformational entropy of the OT ligands, one would expect for the stripe-like NPs a higher gauche to trans ratio for the ligands compared to the patchy one. To measure such conformational changes, we performed SFG measurements also in the C-H stretch region (2800 – 3000 cm^{-1}) of the vibrational spectrum of NPs as shown in Figure S5. The ligand MPA was deuterated so that only the conformation of the OT was probed. Using SFG the alkyl chain conformations can be determined empirically using the amplitude ratio (referred to as d^+/r^+ ratio) of the symmetric methylene (~2850 cm^{-1}, d^+) and the symmetric methyl (~2880 cm^{-1}, r^+) stretch vibrational modes. A value of $d^+/r^+ \ll 1$ is associated with a stretched all-trans alkyl chain conformation, whereas a value of $d^+/r^+ > 1$ indicates that gauche defects dominate the measured vibrational spectrum(5–7). The SFG spectra of both NPs display a different relative amplitude of the d^+ and r^+ mode indicating that the average ligand conformation on the NPs is different. The stripe-like nanoparticle displays a larger d^+/r^+ indicating less stretched alkyl chains.

Work of adhesion measurement from AFM

Images were collected working in the small-amplitude regime in order to map the work(8). The formula proposed in literature to estimate the work of adhesion from the imaging parameters is the following:

$$W_{SL} = a + b\left[\frac{AA_0\left(\sin\varphi - \frac{A}{A_0}\right)}{e^{\frac{-\alpha\sigma}{2}} - e^{\frac{-\alpha(A+\sigma)}{2}}}\right]^2,$$

where A is the working amplitude, A_0 is the free amplitude, φ is the phase shift, σ is the molecular diameter of the liquid and α is the exponential decay length of the density of work of adhesion at the solid-liquid interfaces.

The method does not provide an absolute measurement, but only a relative one that needs to be calibrated through the measurement of contact angle on reference samples. In fact, since the energy dissipation is calculated with respect to the oscillation of the cantilever in the bulk liquid, the intercept of the calibration line is always given by the surface tension of the imaging liquid, therefore only one reference sample is needed for the calibration. We used a film of MPA protected gold nanoparticles as a calibration system for the measurement of the W_{SL} for the patchy NPs. The value of the work of adhesion calculated for these particles was then used as a calibration system for the stripe-like NPs.

To get a reliable comparison of different samples (and hence a good calibration), the exact same A_0 was kept while scanning different samples (differences ≤ 0.05 nm where considered negligible), meanwhile A was kept as constant as possible. Furthermore, images were compared only if collected in the same day and with the same tip.

Each experiment was reproduced twice. A minimum of five nanoparticles per sample were analyzed. Analysis was performed on single particles, extracted from images similar to the ones shown in Figure S7. Data analysis was performed by calculating the average value (and the standard deviation) of $f(A, A_0, \varphi)$ over all the pixels of a single particle masked from an image. This value was then used to calculate the work of adhesion (after the calibration of the system was performed. In the calibration we used 72 mN/m for the surface tension of water).

For every experiment the average value of W_{SL} on different particles and its standard deviation σ was calculated. 5 mN/m were added to each standard deviation in order to take into account the error associated with the calibration ($\sigma^* = \sigma + 5\,mN/m$). The average value for different experiments has been calculated and the final standard deviation has been obtained by propagating the σ^* of the single experiments.

Simulation details

All simulations were carried out using the GROMACS/2018.4 software package(9). Inter-ligand interactions were treated using the OPLS/AA forcefield(10), the SPC/E model(11) was used for water, and the gold surface was described with the GolP model(12). For the patchy and stripe-like surfaces, we started with a box size of ~6 nm x 6 nm in the xy plane, with a height of 30 nm. A gold (111) surface with height 5 nm was placed at the center, and ligands

attached to the surface (the positions of the sulfur atom of each ligand were constrained throughout the simulation, as were all gold atoms other than the image-charge sites(12)). Slabs of water with height 5 nm were placed above and below the ligand surfaces and allowed to condense onto the surface. For the trench surfaces, a similar procedure was followed but with the dimensions of the box 12.1 nm x 2.6 nm in the xy plane. This gives 24 columns of ligands, from which we created trenches containing 0, 1, 2, 4, 8, 12, 16, 20, 22, 23 and 24 columns of MPA ligands (an example of the simulation setup for the trenches is shown in figure S9). Surfaces with random motifs were simulated in boxes with the same dimension as the patchy and striped surfaces, with 20 different sets of random ligands generated, making 40 surfaces. 24 surfaces with tailored motifs were also designed to add to the training set if required. Figure 3A shows an example of the simulation setup for a trench surface after allowing the water slabs to condense.

For every surface, four independent runs were carried out with the initial orientation of ligands about the z axis generated randomly. Simulations were run in the NVT ensemble at 300 K with a timestep of 1 fs and a velocity-rescaling thermostat used to control the temperature(13). After allowing the water slabs to condense onto the ligand surface, the system was equilibrated for 10 ns and statistics were collected over 50 ns. The water-vacuum boundaries produced by expanding the simulation box meant that the pressure was held constant at 0 bar. Long-ranged electrostatics were dealt with using the particle-mesh Ewald (PME) method(14).

The structure of the solvent around the nanoparticle surface was quantified using two properties: the density ρ, obtained by taking a histogram of coordinates of the oxygen atoms in water molecules, and the dipole orientation density $\mu = \langle\cos\theta\rangle\rho$, where $\langle\cos\theta\rangle$ is the average orientation of the water dipole moment with the surface normal pointing away from the gold surface. The surface excesses were obtained by subtracting the predicted histogram from the one observed from simulations, where the prediction was obtained by taking the profiles calculated using surfaces with either full-MPA or full-OT coverage and linearly combining them:

$$\Delta f(z) = f_{obs}(z) - [x_{OT} f_{OT}(z) + x_{MPA} f_{MPA}(z)],$$

where $f_{OT}(z)$ is the profile from the 0-MPA trench and $f_{MPA}(z)$ is the profile from the 24-MPA trench. x_{OT} and x_{MPA} are the fraction of OT and of MPA ligands respectively.

Results on trench configurations

Based on the expectation that the boundary between OT and MPA regions is responsible for the non-additive behavior observed, we ran simulations, as described in the previous section,

on a series of trench geometries, in which the length of the boundary between the two regions is kept constant, but the area of each region differs. If the boundary region alone is responsible for non-additivity, we would expect that the excess properties of interest are independent of the size of the trench, so long as it is large enough that the two boundaries do not "interact" with each other. Figure S11 shows that, indeed, while the surface excesses differ between small and large trench sizes, for large enough trenches the excesses are relatively independent of trench size. This is further borne out by Figure 3A in the main text, in which the local water density is shown in a cut across the trench, and is similarly independent of trench size for large trenches.

When investigating the local density of water, we found that the structure of water in contact with MPA ligands follows one of two regimes, where the two structures are related (in the case of a bare surface) by a 60° rotation, as shown in Figure S10. To obtain the results in Figure 3A, we separated each surface into one or the other type of surface-water structuring, in order to avoid spuriously averaging over two different types of structure and washing-out the local water density.

These trench configurations give a qualitative idea of how the water structure depends on the size of regions of ligand, but for a quantitative picture a wider variety of surface morphologies must be considered.

Nearest-Neighbor Model for Surface Excesses

In order to predict the surface excesses on arbitrary ligands, we developed a model in which an arbitrary excess quantity $\Delta f(z)$ (either $\Delta\rho(z)$ or $\Delta\mu(z)$) is given by the linear combination,

$$\Delta f(z) = \boldsymbol{n} \cdot \boldsymbol{f}(z)$$

$$\Delta f(z) = \sum n_i \, f_i(z),$$

where the index i represents a particular ligand environment, $\mathbf{n} = \{n_i, \ i=1,..,M\}$ is a vector containing the number of ligands n_i of type i, with M the total number of possible environments, and $\boldsymbol{f}(z) = \{f_i(z), \ i=1,..,M\}$, where the functions $f_i(z)$ are to be fitted. This expansion is inspired by the cluster expansion models used to model the thermodynamic properties of multicomponent alloys in terms of local environments(15, 16), with surface excess functions used in our case rather than, e.g., energetics.

We chose to classify ligands based on their nearest neighbors, with the remainder of their surroundings providing a mean-field background. This approach was motivated by the fact that

simulations containing trenches of ligands showed the structure of water to be quite independent of the trench size above the smallest trenches, so that long-ranged effects on the local structure were minimal. The nearest-neighbor environment of a ligand is described by the six ligands surrounding it, meaning that there are 2^7=128 possible ligand plus environment combinations. Of these, many are related by rotational symmetry, and only 26 must be considered, of which the two cases where all six surrounding ligands are identical to the central one were assumed not to contribute to the excess. The remaining 24 possible motifs are shown in Figure S12, along with the fitting functions $\Delta\rho_i(z)$ and $\Delta\mu_i(z)$ corresponding to each.

These functions were obtained by taking a training set of slab systems with the ligand at each site randomly chosen, and for which the excess quantities were computed. In terms of the matrix $\boldsymbol{n}$, whose element $\boldsymbol{n}_{ji}$ gives the number of ligands of type i in the jth training structure, and a vector $\boldsymbol{\Delta f}(z) = \{\Delta f_j(z), j=1,\ldots,N\}$, where $\Delta f_j(z)$ is the surface excess for the jth training structure and N the total number of such structures, the vector of fitting functions is given by,

$$\Delta \boldsymbol{f}(z) = (\boldsymbol{n}^{\boldsymbol{T}}\boldsymbol{n} + \sigma^2\boldsymbol{I})^{-1}\boldsymbol{n}^{\boldsymbol{T}}\boldsymbol{f}(z)$$

with σ^2 a regularization parameter used to avoid overfitting.

Figure S12 shows that the contribution of each motif to a given surface excess is essentially of the same order of magnitude: no particular motif is *a priori* more important than the others in determining the excess. However, the striped surfaces contain both a larger number of environments that give an excess and a larger variety of environments (with several motifs appearing almost exclusively on striped surfaces). These factors lead to more pronounced surface excesses for the striped than for the patchy surfaces.

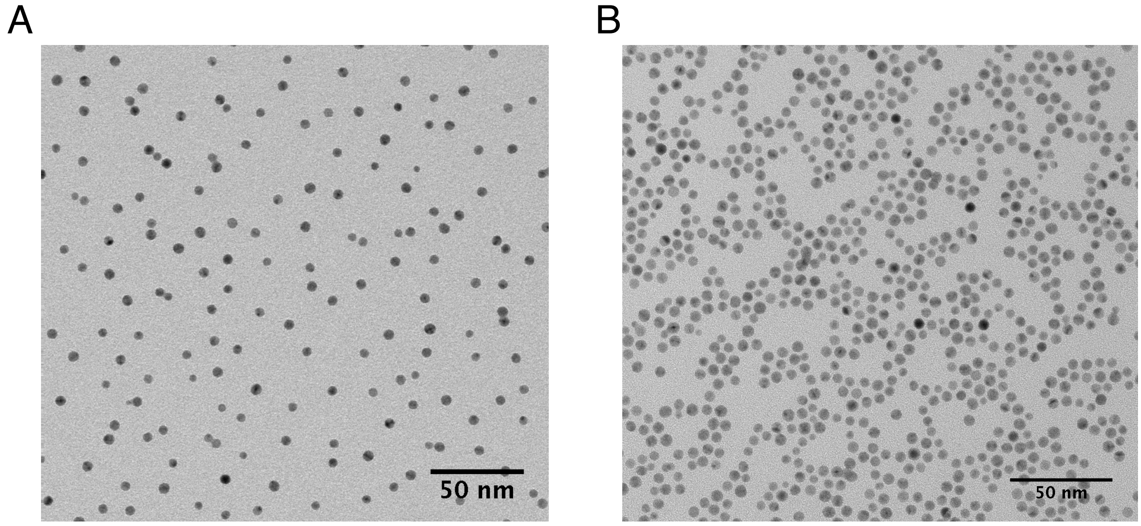

Figure S1 TEM data on the nanoparticles before (A) and after (B) thermal treatment. Based on the counting of more than 500 nanoparticles, the distributions of the core diameter are found to be 5.1 ± 0.6 nm and 5.2 ± 0.7 nm for nanoparticles before and after thermal treatment, respectively.

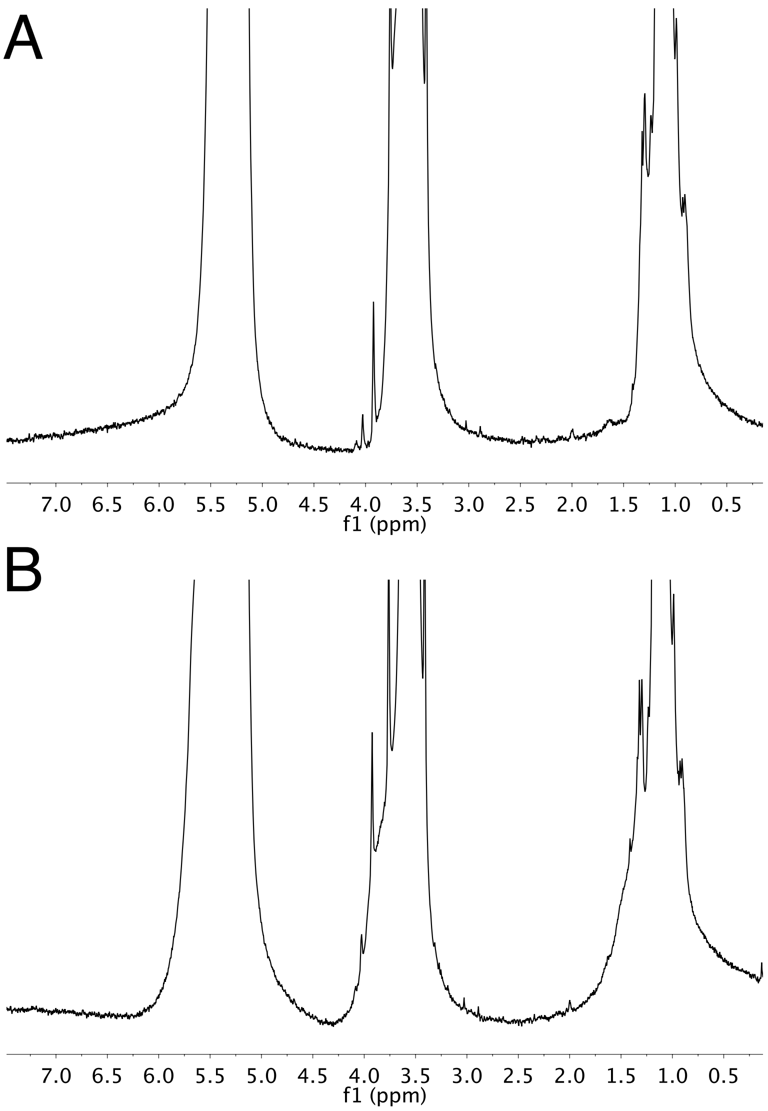

Figure S2 NMR data for nanoparticle before iodine etching. The absence of the sharp peaks between 2.5 and 3.0 ppm indicates that the nanoparticles are free from unbounded small molecules.

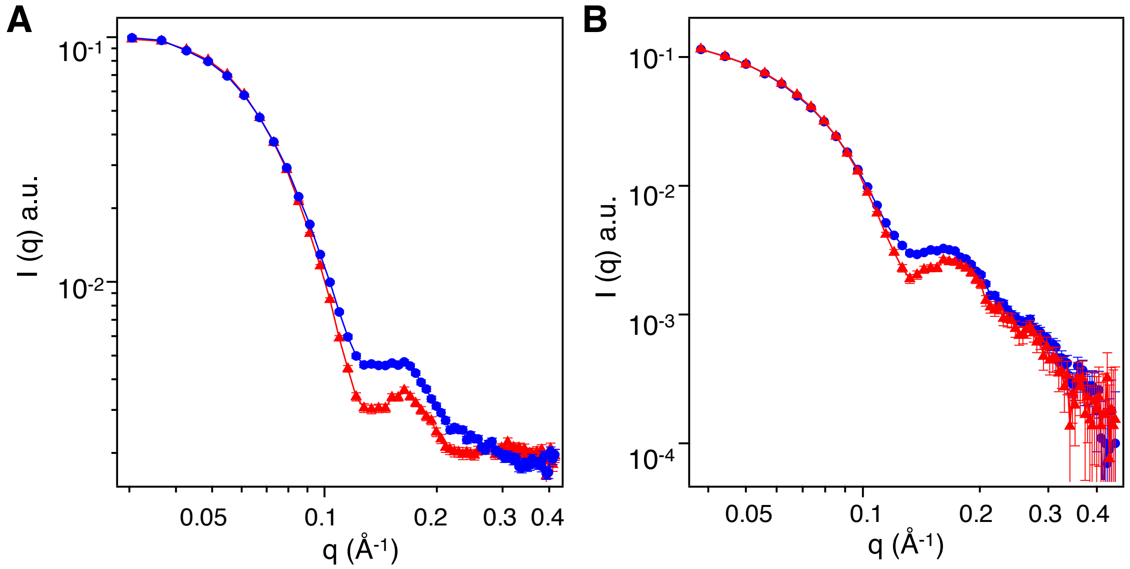

Figure S3 SANS data on the nanoparticles before and after thermal treatment measured at different synchrotron facilities. In both plots, the blue curves stand for nanoparticles before thermal treatment while the red curves stand for samples after thermal treatment. (A) Data collected at MLZ, Germany. (B) Data collected at PSI, Switzerland. The ligands for nanoparticles are the same as shown in the main text, i.e. deuterated MPA and OT.

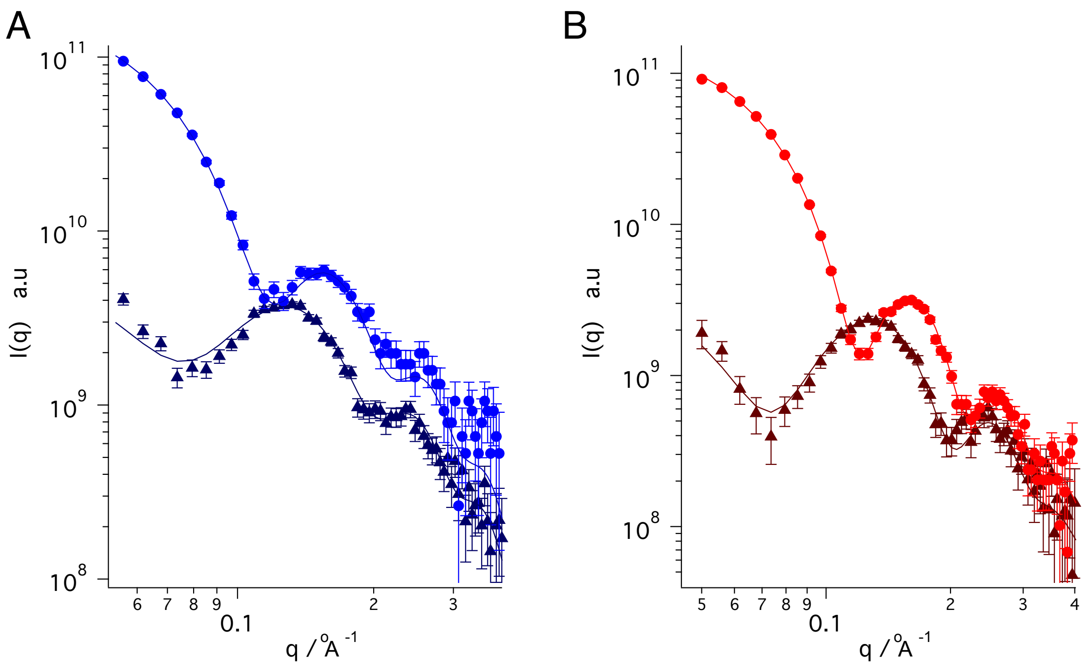

Figure S4 Fitting of the SANS data using MONSA package. In both plots, the full lines are the fits, i.e. form factor of the 3D model generated, while the dots are experimental data. (A) Nanoparticle before thermal treatment. Blue curves stand for SANS measurements in tetrahydrofuran-d8 while dark blue curves stand for SANS measurements in chloroform-d. (B) Nanoparticle after thermal treatment. Red curves stand for SANS measurements in tetrahydrofuran-d8 while dark red curves stand for SANS measurements in chloroform-d.

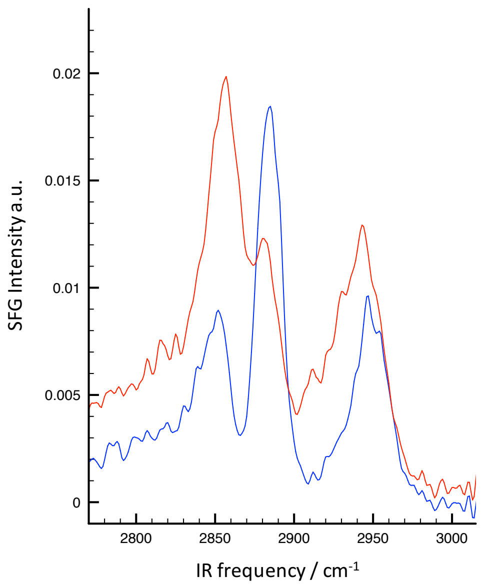

Figure S5 Representative SFG spectra in the C-H region collected in the SSP polarization. The amplitude ratio of the symmetric methylene (~ 2850 cm-1) and the symmetric methyl (~ 2880 cm-1) stretch vibrational modes are significantly different for the patchy and stripe-like particles (respectively shown with a blue and a red curves).

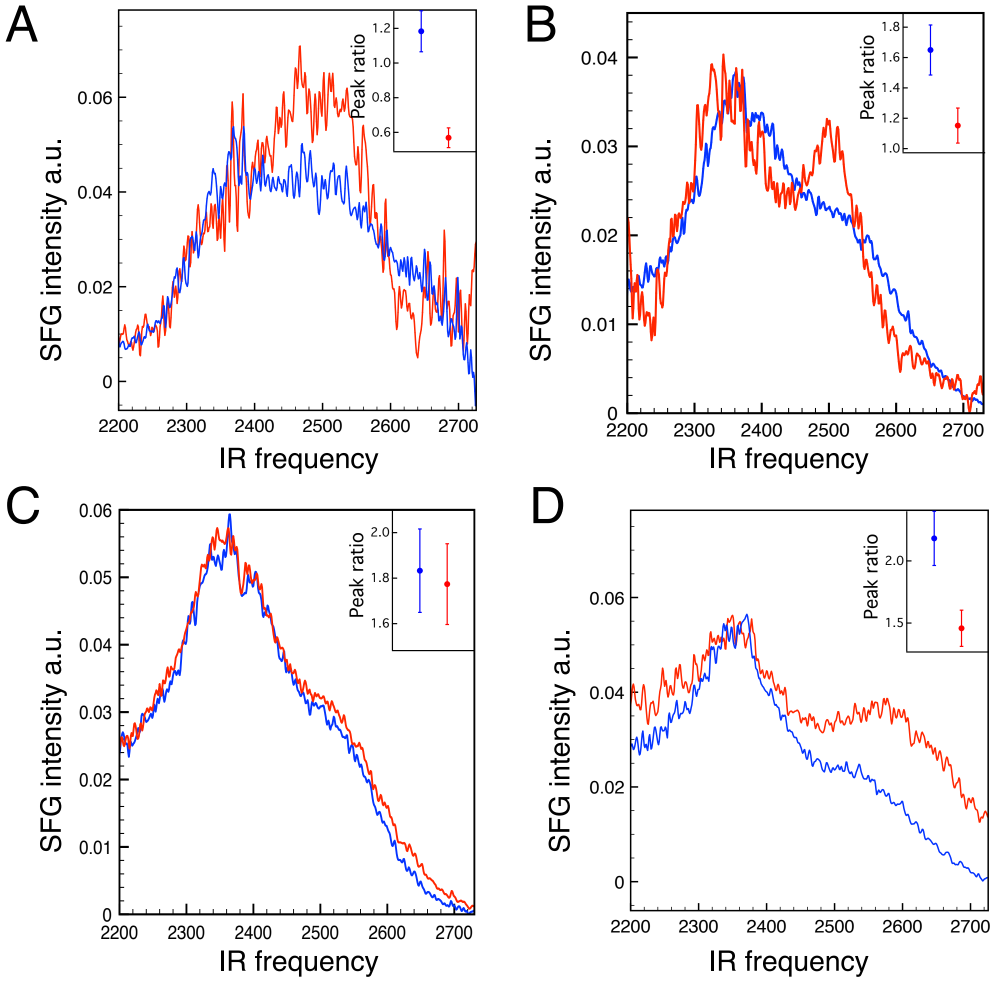

Figure S6 (A)-(D) SFG spectra in the water region, as recorded in four different measurements (Blue: patchy NPs, red: stripe-like NPs) and associated amplitude ratio of the peaks associated with the strong H bond and the weak H bond (insets). To avoid any error from laser fluctuations on different days, we choose to report our data separately. We calculated the peak to peak ratio error of the sum frequency scattering setup on 10 spectra of different samples of sodium dodecylsulfanate stabilized hexadecane nanoemulsions. and the deviation from spectrum to spectrum is less than 10 %. This value is used as an error for the values reported on the above graph. We have never observed an increase in the ratio during an experiment. The fact that during one experiment (C) we did not observe any difference between the two sets of nanoparticles can be explained by the fact that annealing may not have worked perfectly on that occasion. Since in the formation of the film it is likely that similar nanoparticles will

assemble together, it is possible that in this case the laser was focused on an area composed of nanoparticles that were not affected by annealing.

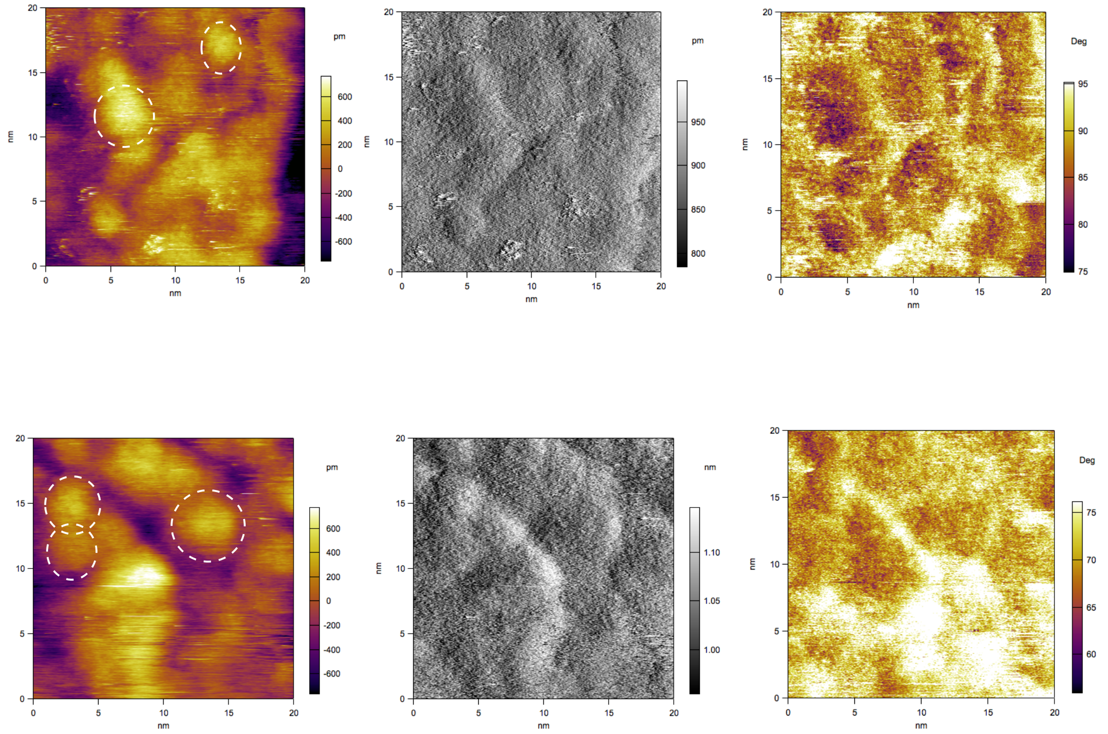

Figure S7 Representative images (in the order: height, amplitude and phase) collected on patchy NPs (top) and on stripe-like NPs (bottom). Circles indicate single particles that have been used for the calculation of W_{SL}. The value of the free phase of oscillation was 95° for the image on top and 76° for the one on the bottom.

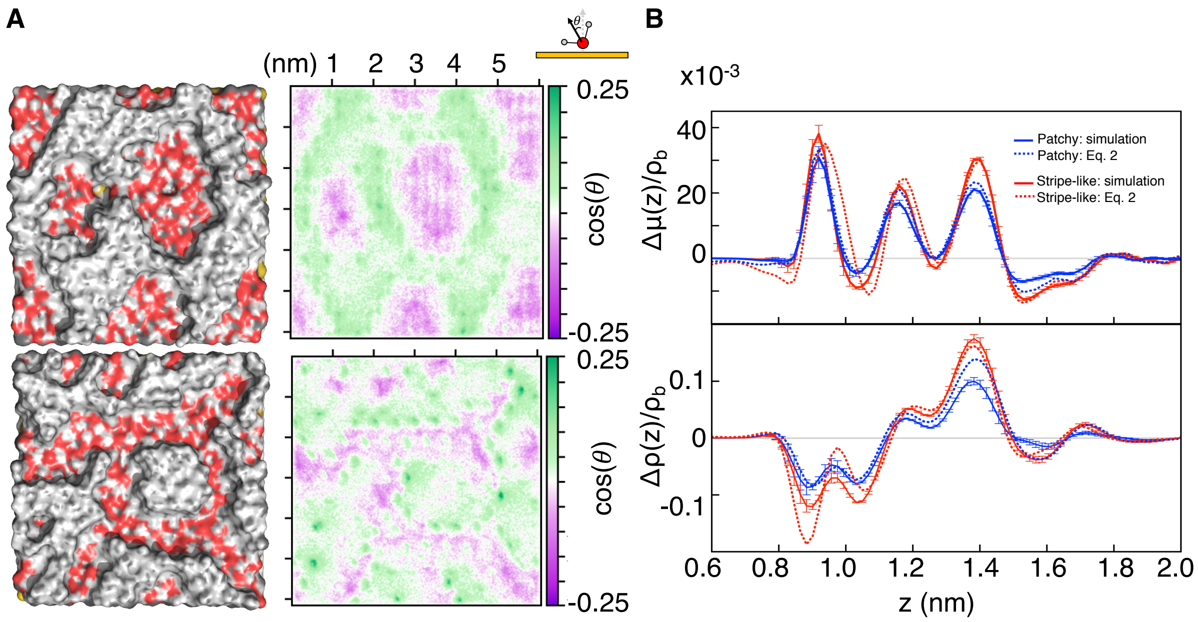

Figure S8 Reproducibility of the results of the simulations. (A) Left: morphology of two additional surfaces after equilibration (patchy on top and stripe-like on the bottom. Water molecules have been removed to show the geometry of the interface. Carbon, oxygen and hydrogen atoms are represented with grey, red and white colors respectively. Right: corresponding 2D histogram of the average dipole orientation density, μ, above the surfaces (averaged over four different realizations). Areas on top of the MPA patches look mostly purple (dipole pointing towards the surface) and areas on top of the OT patches look mostly white (dipole parallel to the surface). Green regions (dipole pointing out from the surface) are visible on top of the OT-MPA interfaces, with darker green visible on the stripe-like morphology. (B) Deviation from the additive assumption of the average dipole orientation density, Δμ, (top) and density profile of water, Δρ (bottom), as a function of the distance from the gold surface (z). Both result of the molecular dynamics simulation and the prediction from equation 2 are shown: good agreement between them is visible.

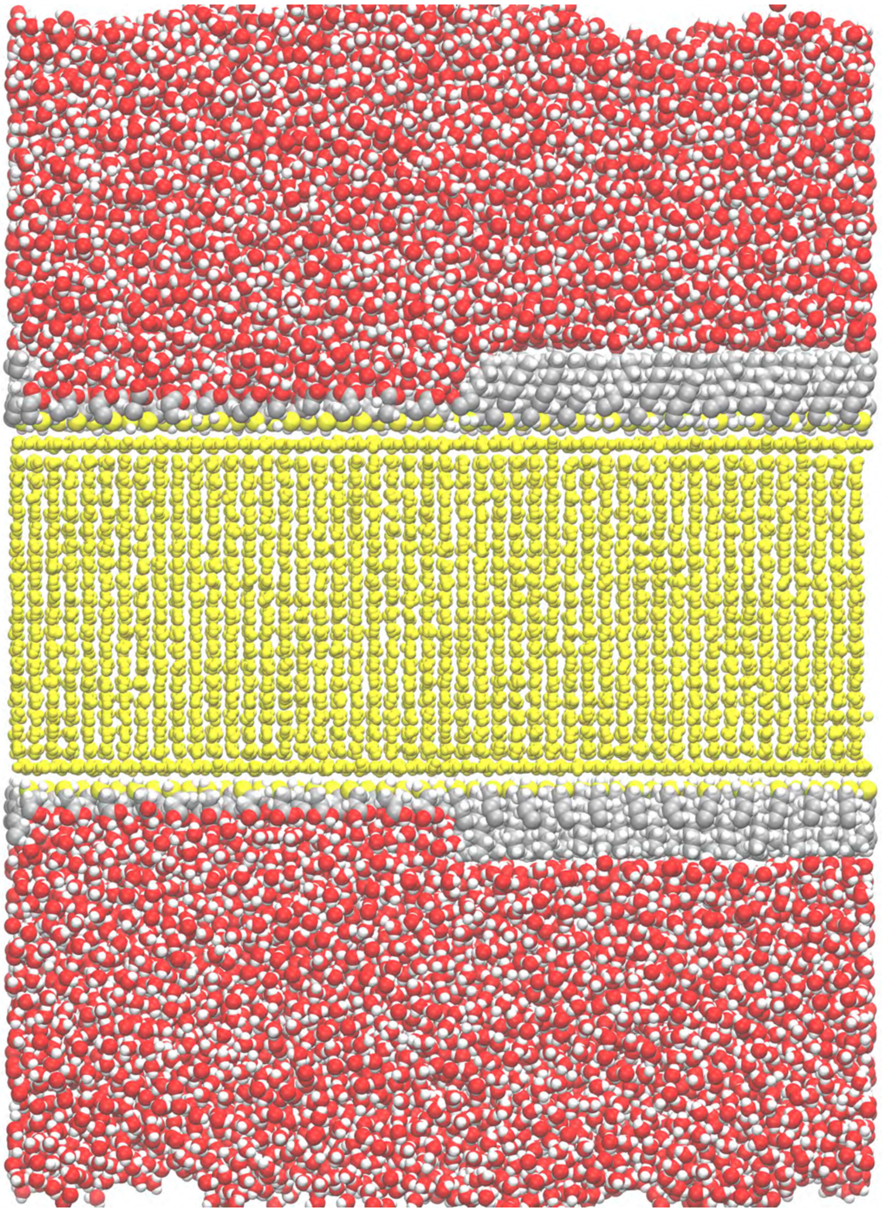

Figure S9 Illustration of the simulation setup for a trench configuration with 12 MPA and 12 OT ligands. Gold atoms are shown in yellow, carbon in grey, oxygen in red and nitrogen in white.

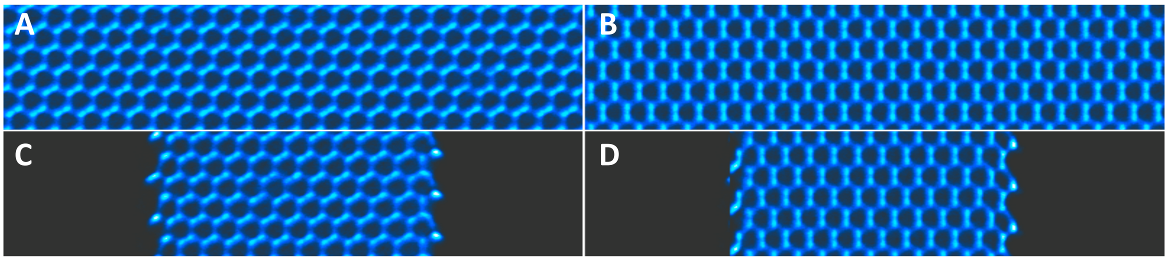

Figure S10 Water density on MPA trenches. Top row: two types of surface structure for a surface with 24 rows of MPA ligands; these are rotationally identical. Bottom row: (C) and (D) show the surface structure of water on top of the MPA ligands, equivalent to the structures in (A) and (B) respectively. Because of the presence of a region of OT ligands, these two are no longer equivalent.

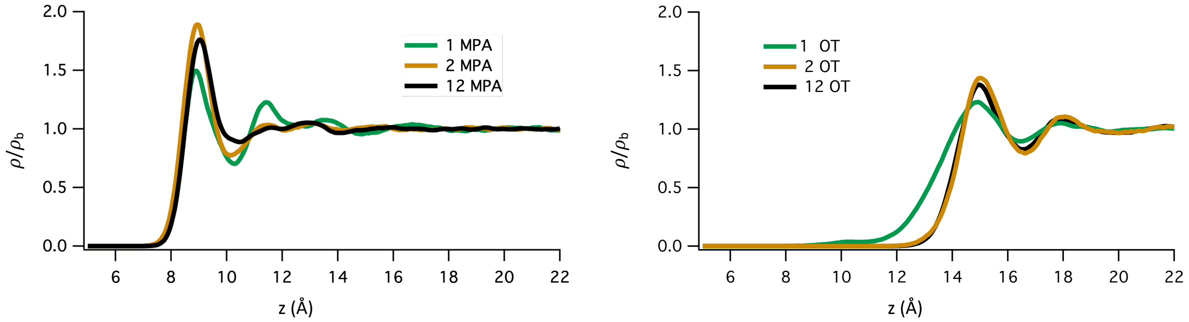

Figure S11 Density profile of water as a function of the distance from the gold surface at the center of a trench. Values measured at the center of the MPA and OT trenches are shown on the left-hand side and right-hand side respectively. In both cases the density of water on the 2 molecules thick trench (brown) is comparable with the one on the center of the 12 molecule thick trench (black). On the contrary the density of water on the single-molecule thick trench (green) looks significantly different from the trench that is 2 molecules thick.

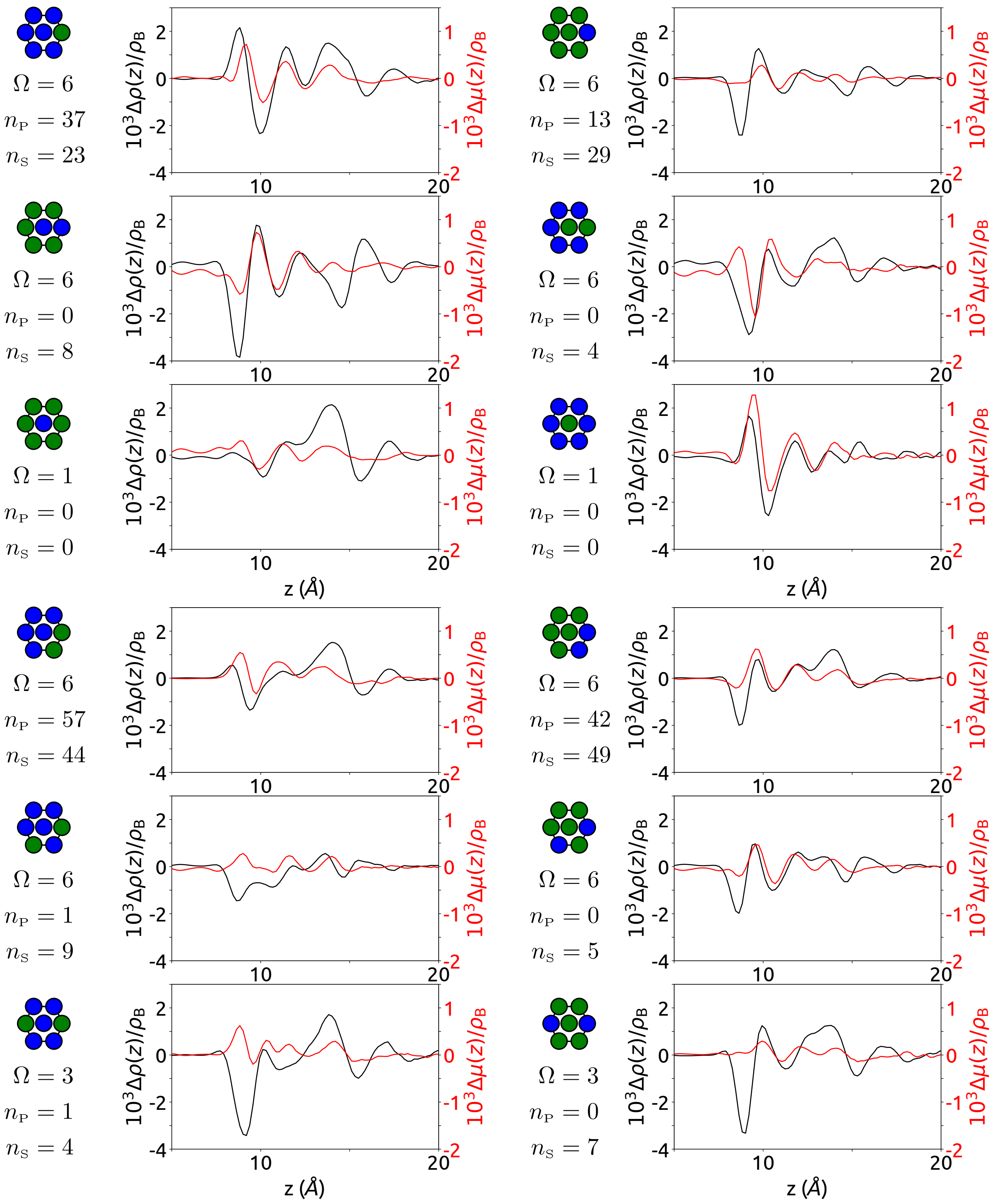

Ω = 6
n_P = 37
n_S = 23
Ω = 6
n_P = 13
n_S = 29
Ω = 6
n_P = 0
n_S = 8
Ω = 6
n_P = 0
n_S = 4
Ω = 1
n_P = 0
n_S = 0
Ω = 1
n_P = 0
n_S = 0
Ω = 6
n_P = 57
n_S = 44
Ω = 6
n_P = 42
n_S = 49
Ω = 6
n_P = 1
n_S = 9
Ω = 6
n_P = 0
n_S = 5
Ω = 3
n_P = 1
n_S = 4
Ω = 3
n_P = 0
n_S = 7
10^3Δρ(z)/ρ_B
10^3Δμ(z)/ρ_B
z (Å)

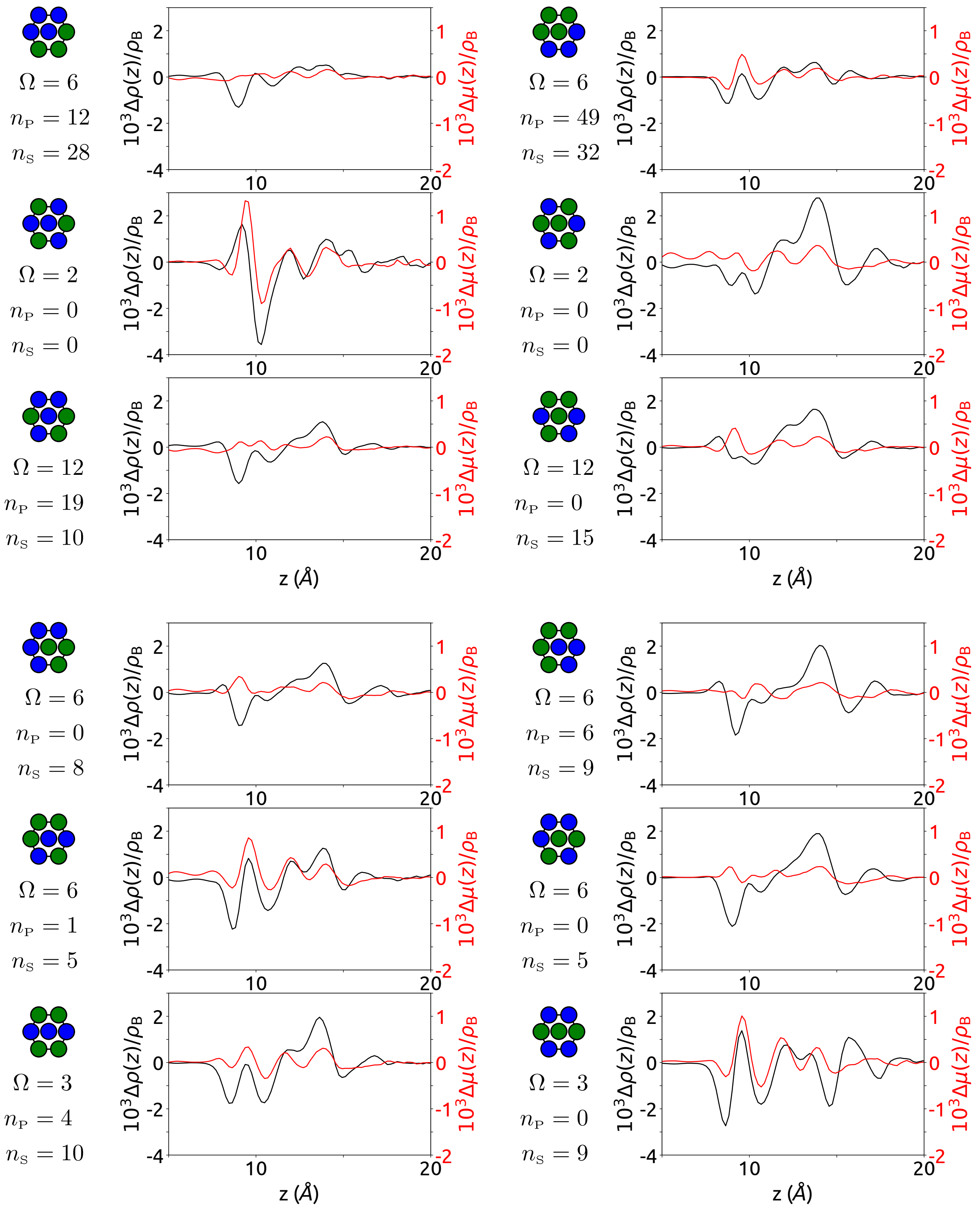

Figure S12 Excess terms associated to all the possible nearest neighbors patterns. OT and MPA ligands are shown in blue and green respectively. **Ω** stands for the degeneracy of the pattern. $\boldsymbol{n_P}$ and $\boldsymbol{n_S}$ are the number of times the pattern is observed in the patchy and stripe-like surfaces shown in figure 2D, respectively.

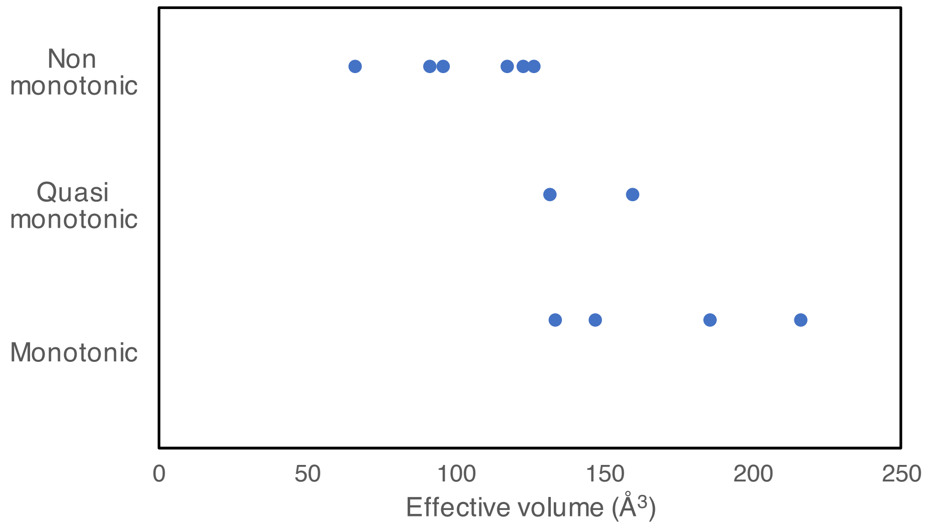

Figure S13 Re-analysis of the solubility profiles of similar MPA-OT nanoparticles as previously reported(17). The solvents are classified into three categories depending on whether the nanoparticles show monotonic dependence of solubility on ligand shell composition. The solvents are then arranged using their effective molecular volume. In general, solvents that give non-monotonic solubility behavior of the nanoparticles have smaller effective molecular volume, while solvents that give monotonic solubility behavior of the nanoparticles have larger effective molecular volume.

Table S1 Summary of the characterization of the nanoparticles.

	**Patchy NP**	**Stripe-like NP**
**Core diameter**	4.9 ± 0.6 nm	4.9 ± 0.6 nm
**MPA: OT ratio**	55%: 45%	54%: 46%
**Ligand weight percentage**	5.9%	5.5%
**MPA domain thickness**	2.1 ± 0.6 nm	1.3 ± 0.3 nm

Table S2. Values of water contact angle measured on films of NPs and corresponding work of adhesion (calculated by the Young-Dupré equation, using 72 mN/m for the surface tension of water). The error on the contact angle reported is the standard deviation of the measured values, while the error on W_{SL} is obtained by propagating the one on contact angle.

Sample	Contact Angle	Work of adhesion
**Patchy Nanoparticle**	76 ± 3 °	89 ± 4 mN/m
**Stripe-like Nanoparticle**	85 ± 3 °	79 ± 4 mN/m
**MPA Nanoparticle**	38 ± 1 °	128 ± 1 mN/m
**OT Nanoparticle**	106 ± 5 °	52 ± 6 mN/m

Table S3. Contact angle values obtained on an additional set of MPA-OT nanoparticles with ligand ratio 70:30 (as obtained by NMR) and core size 4.1 ± 0.5 nm (as obtained by TEM). These measurements confirm the fact that the wetting of the nanoparticles is affected by the shell morphology.

Sample	Contact Angle	Work of adhesion
**Before annealing**	72 ± 2 °	94 ± 3 mN/m
**After annealing**	83 ± 1 °	81 ± 1 mN/m
**Difference**	11 ± 1 °	13 ± 1 mN/m

Table S4. Values of surface tension (γ), density (d), molar weight (M) and measured contact angle of various solvents on the two nanoparticle films.

Solvents	γ (mN/m)	d (g/ml)	M (g/mol)	$V_{effective}$‡ (Å^3)	θ_{Patchy} (°)	$\theta_{Stripe\text{-}like}$ (°)
Water(18)	72.8	1.00	18.0	30.0	70.1 ± 2.7	78.6 ± 2.9
Dichlorobenzene(18)	33.6	1.31	147.0	187.0	30.8 ± 0.8	36.1 ± 2.0
Chloroform(18)	27.5	1.49	119.4	133.0	16.6 ± 1.7	16.8 ± 2.4
Ethylene glycol(18)	47.7	1.11	62.1	92.6	52.2 ± 2.3	64.9 ± 3.3
DMSO(18)	44.0	1.10	78.1	118.0	44.7 ± 3.8	52.0 ± 3.7
Perfluorodecalin(19)	19.0	1.91	462.1	402.3	25.0 ± 1.3	27.7 ± 3.0
5CB(20)	27.9	1.00	249.4	411.0	46.3 ± 2.2	50.0 ± 1.8
Glycerol(18)	63.4	1.25	92.1	122.4	71.2 ± 4.1	87.2 ± 4.5
Hexadecane(19)	27.5	0.77	226.4	486.6	14.7 ± 2.2	25.5 ± 2.6
Isopropanol(18)	23.0	0.79	60.1	127.2	0*	6.8 ± 3.3
Formamide(21)	58.9	1.13	45.0	66.0	46.1 ± 2.0	51.6 ± 2.7
Perfluorohexane(19)	11.7	1.67	338.0	336.4	4.8 ± 1.4	5.8 ± 2.6

* $\theta = 0°$ is approximated for solvents that wet almost completely the nanoparticle film.

‡ The effective molecular volume is calculated by dividing the molar volume by Avogadro's number, i.e., $V_{effective} = \frac{M}{d * N_A}$

SI References

1. A. Centrone, Y. Hu, A. M. Jackson, G. Zerbi, F. Stellacci, Phase separation on mixed-monolayer-protected metal nanoparticles: A study by infrared spectroscopy and scanning tunneling microscopy. *Small* **3**, 814–817 (2007).

2. Z. Luo, *et al.*, Quantitative 3D determination of self-assembled structures on nanoparticles using small angle neutron scattering. *Nature Communications* **9**, 1343 (2018).

3. D. I. Svergun, Restoring low resolution structure of biological macromolecules from solution scattering using simulated annealing. *Biophysical journal* **76**, 2879–86 (1999).

4. D. I. Svergun, Determination of the regularization parameter in indirect-transform methods using perceptual criteria. *Journal of Applied Crystallography* **25**, 495–503 (1992).

5. P. Guyot-Sionnest, J. H. Hunt, Y. R. Shen, Sum-frequency vibrational spectroscopy of a Langmuir film: Study of molecular orientation of a two-dimensional system. *Physical Review Letters* **59**, 1597–1600 (1987).

6. O. Esenturk, R. A. Walker, Surface vibrational structure at alkane liquid/vapor interfaces. *The Journal of Chemical Physics* **125**, 174701 (2006).

7. E. Tyrode, J. Hedberg, A Comparative Study of the CD and CH Stretching Spectral Regions of Typical Surfactants Systems Using VSFS: Orientation Analysis of the Terminal CH3 and CD3 Groups. *The Journal of Physical Chemistry C* **116**, 1080–1091 (2012).

8. K. Voïtchovsky, J. J. Kuna, S. A. Contera, E. Tosatti, F. Stellacci, Direct mapping of the solid–liquid adhesion energy with subnanometre resolution. *Nature Nanotechnology* **5**, 401 (2010).

9. M. J. Abraham, *et al.*, GROMACS: High performance molecular simulations through multi-level parallelism from laptops to supercomputers. *SoftwareX* **1–2**, 19–25 (2015).

10. M. J. Robertson, J. Tirado-Rives, W. L. Jorgensen, Improved Peptide and Protein Torsional Energetics with the OPLS-AA Force Field. *Journal of Chemical Theory and Computation* **11**, 3499–3509 (2015).

11. H. J. C. Berendsen, J. R. Grigera, T. P. Straatsma, The missing term in effective pair potentials. *The Journal of Physical Chemistry* **91**, 6269–6271 (1987).

12. I. F., D. F. R., M. E., C. S., GolP: An atomistic force-field to describe the interaction of proteins with Au(111) surfaces in water. *Journal of Computational Chemistry* **30**, 1465–1476 (2008).

13. G. Bussi, D. Donadio, M. Parrinello, Canonical sampling through velocity rescaling. *J. Chem. Phys.* **126**, 014101 (2007).

14. T. Darden, D. York, L. Pedersen, Particle mesh Ewald: An N·log(N) method for Ewald sums in large systems. *The Journal of Chemical Physics* **98**, 10089–10092 (1993).

15. J. Z. Liu, A. van de Walle, G. Ghosh, M. Asta, Structure, energetics, and mechanical stability of Fe-Cu bcc alloys from first-principles calculations. *Phys. Rev. B* **72**, 144109 (2005).

16. A. Díaz-Ortiz, H. Dosch, R. Drautz, Cluster expansions in multicomponent systems: precise expansions from noisy databases. *J. Phys.: Condens. Matter* **19**, 406206 (2007).

17. A. Centrone, *et al.*, The role of nanostructure in the wetting behavior of mixed-monolayer-protected metal nanoparticles. *Proceedings of the National Academy of Sciences* **105**, 9886–9891 (2008).

18. J. Shen, *et al.*, Liquid Phase Exfoliation of Two-Dimensional Materials by Directly Probing and Matching Surface Tension Components. *Nano Letters* **15**, 5449–5454 (2015).

19. A. Luís, *et al.*, Influence of Nanosegregation on the Surface Tension of Fluorinated Ionic Liquids. *Langmuir* **32**, 6130–6139 (2016).

20. M. G. J. Gannon, T. E. Faber, The surface tension of nematic liquid crystals. *Philosophical Magazine A* **37**, 117–135 (1978).

21. B. Jańczuk, W. Wójcik, A. Zdziennicka, Determination of the components of the surface tension of some liquids from interfacial liquid-liquid tension measurements. *Journal of Colloid And Interface Science* **157**, 384–393 (1993).
